# Capillary Blood Gas Predicts Risk of Intensive Care in Children with Bronchiolitis

**DOI:** 10.3390/children8080719

**Published:** 2021-08-23

**Authors:** August Wrotek, Małgorzata Kobiałka, Teresa Jackowska

**Affiliations:** 1 Department of Pediatrics, Centre of Postgraduate Medical Education, Marymoncka 99/103, 01-813 Warsaw, Poland; kobialkm@bielanski.med.pl; 2 Department of Pediatrics, Bielanski Hospital, Cegłowska 80, 01-809 Warsaw, Poland

**Keywords:** bronchiolitis, RSV, capillary blood gas, intensive care, acidosis, hypercapnia

## Abstract

Background: Bronchiolitis may result in respiratory failure diagnosed with arterial blood gas (ABG). ABG is not routinely performed in general paediatric wards but is closely reflected by capillary blood gas (CBG). We sought to assess the usefulness of CBG results in prediction of intensive care unit (ICU) transfer, antibiotic treatment, and length of stay in children hospitalized due to bronchiolitis. Methods: The optimal cutoff values were estimated with an ROC analysis, while a multiple regression model calculated the odds of an ICU transfer, prolonged hospitalization, and antibiotic treatment related with hypercapnia (pCO_2_ ≥ 45 mmHg) and acidosis (pH ≤ 7.35). The correlation between the CBG (pH, pCO_2_, and SatO_2_) and the clinical/laboratory parameters (breath rate, heart rate, pulse oximetry, white blood cells, CRP, and procalcitonin) was calculated. Results: The CBG was performed in 485 children aged 8 days–22 months (median 2 months). The pCO_2_ was significantly higher in ICU transferred patients (median 44.8 mmHg vs. 36.2 mmHg, *p* < 0.01), and showed AUC = 0.773, (95% CI: 0.638–0.907, *p* < 0.01) for ICU transfer (67% sensitivity, 82% specificity, 10.8% positive and 98.7% negative predictive value at cutoff 41.8 mmHg). Hypercapnia (OR = 6.63, 95% CI: 2.15–20.46, *p* < 0.01) and acidosis (OR = 5.01, 95% CI: 1.26–19.9, *p* = 0.022) predicted the ICU transfer independently. The CBG parameters were not related to prolonged hospitalization or antibiotic treatment, and showed only a weak and clinically irrelevant correlation with other laboratory and clinical parameters. Conclusions: Acidosis and hypercapnia indicate patients at risk of an ICU transfer, and the pCO_2_ levels (including values lower than hypercapnia) seem to be a promising marker in ICU risk assessment.

## 1. Introduction

Bronchiolitis is an inflammatory process defined as the first episode of wheezing in children under 24 months of age and is a major single cause of paediatric hospitalizations in infants [1]. In the vast majority of cases, bronchiolitis is caused by the respiratory syncytial virus (RSV), which is responsible for roughly 80% of cases [2,3]. Except for monoclonal antibodies administration, which is passive immunoprophylaxis, there is no registered active immunoprophylactic measure against RSV (i.e., vaccine) at the moment, although clinical trials are being conducted [4]. The clinical course of bronchiolitis may vary from a self-limiting disease to a more severe patient condition, including the risk of apnea or respiratory failure. The risk of intensive care unit (ICU) treatment is estimated at approximately 3% of emergency department patients [5]. The laboratory criteria of respiratory failure are based upon an arterial blood gas (ABG) analysis [6]; thus, ABG is considered a gold standard [7]. Nevertheless, due to its invasive character, dependence on a physician, and high risk of complications, which include haemorrhage and a risk of thrombosis or aneurysm, it is not widely accepted and used in the field of paediatrics, except for the intensive care unit setting [7]. Moreover, repeated sampling is difficult, unless an indwelling catheter is present [8]. There are two possible alternatives to ABG: venous (VBG) or capillary blood gas (CBG). A correlation between the arterial and capillary blood gas has been shown in several studies; although the studies were set in intensive care units [7,9,10], the results may be extended to the group of children hospitalized because of bronchiolitis, since one of the most important risks related to bronchiolitis is respiratory failure and an ICU transfer. The British National Institute for Health and Care Excellence (NICE) guidelines on the management of bronchiolitis advise that capillary blood gas may be considered in patients who deteriorate severely or are at an impending risk of respiratory failure [11]. A suspicion of respiratory failure should be raised when there are signs of exhaustion (e.g., decreased respiratory work or listlessness), recurrent episodes of apnea or if oxygen saturation cannot be maintained with oxygen supplementation [11]. Nevertheless, there is only a limited number of guidelines that take CBG into consideration, while some (e.g., Canadian Paediatric Society) mention blood gas if there is a concern of respiratory failure, without specifying which type of blood analysis (arterial, venous, or capillary) should be performed [12]. Nonetheless, CBG is generally absent in the vast majority of guidelines, including those by the American Academy of Pediatrics, Australasian or local Polish recommendations [1,13,14]. In this retrospective cohort study, we intended to assess the usefulness of CBG in the prediction of the need for an ICU transfer in hospitalized children. A correlation between the CBG parameters and disease severity was sought, and included laboratory and clinical parameters, antibiotic treatment, and the length of stay (LOS).

## 2. Materials and Methods

Children hospitalized due to bronchiolitis in the period from January 2010 to June 2018 who had their CBG performed on admission were eligible for the study. Electronic medical charts of patients hospitalized at the paediatric ward of the Bielanski Hospital, Warsaw were retrospectively reviewed. The search included the final diagnoses of bronchiolitis (J21 with its extensions) according to the International Classification of Diseases, Tenth Revision (ICD-10). The diagnosis of bronchiolitis was based upon clinical signs and symptoms according to the definition by the American Academy of Pediatrics 2006 guidelines, and the corresponding Polish guidelines, which included: a prodromal period of upper respiratory tract infection (rhinitis, cough) followed by cough, abnormalities on auscultation (wheezing and/or crackles), and difficulties in breathing (use of accessory muscles/chest recession, or nasal flaring) [14,15]. The diagnoses were made by an attending physician, and patients’ medical charts were reviewed to verify correctness of the diagnosis. Exclusion criteria consisted of immunodeficiency (congenital or acquired or drug-related), lack of full knowledge of the clinical course of the disease (e.g., a discharge at parents’/tutors’ request). The anonymized data were retrieved and contained demographical data (age and gender), pregnancy history (number of pregnancies, history of miscarriage, multiple/singleton pregnancy, diseases during pregnancy, nicotine exposition, and mother’s age), data regarding delivery (week of pregnancy, weight, and Apgar score), history of breastfeeding, vaccination, and comorbidities (cardiopulmonary diseases, immunodeficiency, and drug administered to a child). A recent history of the disease data included: duration of signs/symptoms between the first presentation and hospital admission, presence of particular signs/symptoms (fever, rhinitis, cough, dyspnea, apnea, and problems with feeding), clinical examination at admission (breath rate—BR, heart rate—HR, oxygen saturation measured by pulse oximetry, wheezing, crackles, use of accessory respiratory muscles, chest recession, and nasal flaring), and laboratory parameters at admission (white blood cells count—WBC, absolute neutrophil count—ANC, C-reactive protein—CRP, and procalcitonin—PCT), as well as the presence of RSV in an upper respiratory tract sample (aetiology was confirmed with a rapid antigen test and/or polymerase-chain reaction, PCR). The CBG was performed on admission in each enrolled patient; a capillary blood sample was taken, after site disinfection, without prior warming of the finger. The initial puncture with a needle was performed, then a tube was contacted with the incision and capillary blood was obtained; each sample was verified regarding the presence of air bubbles and analysed immediately with the use of the Roche Cobas b 121 and b 221 analyser (Roche Diagnostics Ltd., Rotkreuz, Switzerland) until 26 January 2016 and with the use of the RAPIDLab 348EX Blood Gas System by Siemens (Siemens Healthcare Diagnostics, Marburg, Germany) after 27 January 2016. All the procedures were performed in accordance with the manufacturer’s instructions. The following CBG parameters were analysed: pH, partial carbon dioxide pressure (pCO_2_) and oxygen saturation (SatO_2_).

The major endpoint was the ICU transfer, while the secondary endpoints were prolonged hospitalization and antibiotic treatment; additionally, a correlation between the CBG parameters and continuous clinical and laboratory parameters as well as internal correlation between the CBG parameters was assessed.

Data distribution was analysed with the Kolmogorov Smirnov test, and the data was presented as a mean +/− standard deviation (SD) for normally distributed data, or as a median and an interquartile range (IQR) for not-normally distributed data. Statistical significance was verified with the corresponding parametric (Student’s *t*-test) or non-parametric (Mann–Whitney U) test.

A receiver-operating characteristic (ROC) curve analysis with the use of the Youden index was performed in order to calculate the optimal cutoff values for continuous data (CBG parameters) in prediction of binary endpoints (ICU transfer, antibiotic treatment, and prolonged length of stay defined as the length of stay over the median value). The results were presented as the area under the curve (AUC) with a 95% confidence interval (95% CI), and sensitivity, specificity, as well as positive and negative predictive value (PPV and NPV, respectively) for the calculated cutoff values. A multiple regression model was created in order to compute the risk of an ICU transfer related to acidosis and hypercapnia. For this purpose, acidosis was defined as a pH equal to or less than 7.35, whereas the hypercapnia as a pCO_2_ of 45 mmHg or more. The correlation between continuous data was calculated with the Spearman’s rank correlation test, and the statistically significant correlation coefficients (rho) were presented.

A *p* value under 0.05 was considered statistically significant. The statistical analysis was conducted with Statistica 13.1 software (Statsoft, Tulsa, OK, USA). Confidence intervals for sensitivity, specificity, PPV and NPV were calculated with the use of the diagnostic test evaluation calculator available at https://www.medcalc.org/calc/diagnostic_test.php (accessed on 31 January 2021).

The study was conducted in accordance with the Helsinki Declaration with later amendments, and granted permission by the local Ethics Committee at the Centre of Postgraduate Medical Education in Warsaw (permission number 141/PB/2020). Patient’s and parent’s/tutor’s consent was waived due to the retrospective character.

## 3. Results

During the analysed period, there were 534 hospitalizations due to bronchiolitis and finally the group consisted of 485 (91%) children (275 boys, 210 girls) who had the CBG calculated on admission. Patients’ age varied from 8 days to 22 months (median 2 months), and the group included 56 neonates and 7 children older than 12 months. The majority of cases (83.5%, 405 out of 485) was caused by the respiratory syncytial virus. Fifteen children required intensive care (13 in the RSV group and 2 in the non-RSV group). Children transferred to the ICU were younger (median 1 vs. 2.13 months, *p* < 0.01), and the only significant difference among the analysed clinical and laboratory parameters (except the BCG) was observed in the breath rate (64.5 vs. 60 per minute, *p* = 0.023) (Table 1). The partial carbon dioxide pressure was significantly higher in patients who required ICU transfer (median 44.8 mmHg vs. 36.2 mmHg, *p* < 0.01), while the other analysed CBG parameters remained insignificant (Table 1).

**Figure 1:**
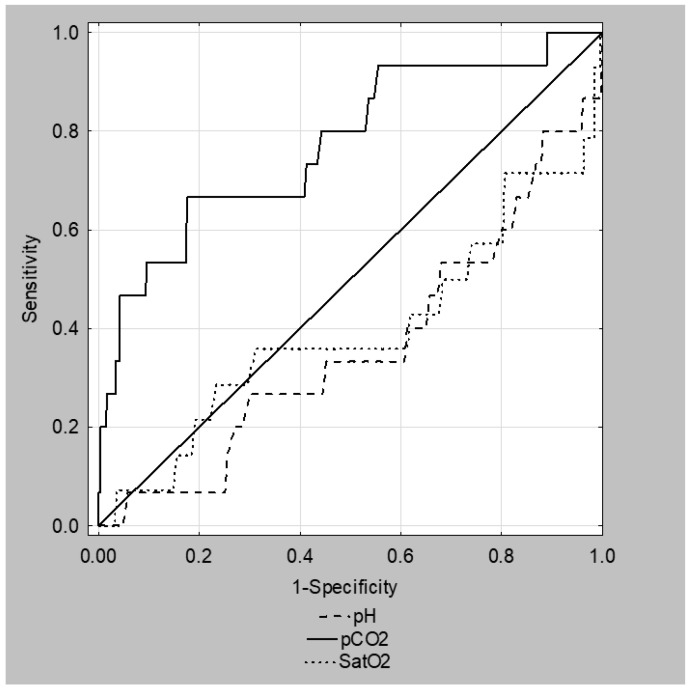
ROC curves for pH, pCO_2_, and SatO_2_ in prediction of an ICU transfer.

In an ROC analysis, the pCO_2_ showed the most promising value in the prediction of an ICU transfer with the AUC of 0.773 (95% CI: 0.638–0.907, *p* < 0.01), while the other CBG parameters remained insignificant (Figure 1). The cutoff value of 41.8 mmHg (95% CI: 35.6–41.8) showed a 67% sensitivity (95% CI: 38.4–88.2%), and an 82% specificity (95% CI: 78.6–85.7%), with a 10.8% positive predictive value (95% CI: 7.4–15.3%), and a 98.7% negative predictive value (95% CI: 97.4–99.4%). No statistical significance was found in terms of the antibiotic treatment or prolonged length of stay for any of the CBG parameters.

In a multiple logistic regression model, patients with hypercapnia were at a higher risk of an ICU transfer (aOR = 6.63, 95% CI: 2.15–20.46, *p* < 0.01), but not the risk of an antibiotic treatment or a longer hospital stay. Acidosis was also related to a higher risk of an ICU transfer (aOR = 5.01, 95% CI: 1.26–19.9, *p* = 0.022), and similarly to hypercapnia, acidosis did not correlate with the increased risk of an antibiotic therapy or a prolonged length of stay (Table 2). The aforementioned breath rate (which differed between those transferred and those not transferred to the ICU) also correlated inversely with the age (rho = −0.15). In a multiple regression model, tachypnoea (according to the World Health Organization definition per age, i.e., over 60 breaths/minute in neonates, >50 in 2–12 month olds, >40 in 1–5 year olds) was not related to an increased risk of an ICU transfer.

A weak correlation between the CBG parameters and the patients’ age was observed: rho(pH) = 0.15, rho(pCO_2_) = −0.46, rho (SatO_2_) = 0.35; thus, in a multiple regression model, a correction for age was been made. The CBG parameters showed only a weak and clinically irrelevant correlation with other laboratory and clinical parameters. The pH correlated with the WBC (rho = 0.09), ANC (rho = 0.12) and the CRP (rho = 0.14) but not with the PCT; it also correlated with the pulse oximetry oxygen saturation (rho = 0.11) and inversely with the HR (rho = −0.11). The pCO_2_ inversely correlated with the WBC (rho = −0.22), ANC (rho = −0.23), and CRP (rho = −0.11) but not with the PCT, and among the clinical parameters, a positive correlation was observed with the BR (rho = 0.18) and the HR (rho = 0.17) and an inverse correlation with the pulse oximetry (rho = −0.24). The SatO_2_ correlated positively only with the ANC (rho = 0.14) and pulse oximetry (rho = 0.23) and inversely with the BR (rho = −0.15) and the HR (rho = −0.21). Only a weak correlation (rho = 0.23) between the oxygen blood saturation in pulse oximetry and the CBG SatO_2_ was observed. No correlation of any CBG parameter was shown regarding the length of stay. The internal correlation between the parameters was as follows: rhopH-pCO_2_ = −0.61, rhopH-SatO_2_ = 0.36, rhopCO_2_-SatO_2_ = −0.55 (Table 3).

## 4. Discussion

Our study showed that the CBG, especially the pCO_2_, may be a useful tool in predicting the risk of an ICU transfer. We need to emphasize that blood gas parameters can easily be measured in a capillary blood sample, which is a more available and acceptable method than the ABG, and acidosis and hypercapnia present significant value in assessing the risk of an ICU transfer. Moreover, in our series of patients, elevated pCO_2_ showed a promising AUC (=0.773) and returned a very high negative predictive value; importantly, the optimal cutoff point in the ROC analysis was established at a lower value than the hypercapnia definition (in this group: 41.8 mmHg); patients with a pCO_2_ under this limit had a 98.7% chance of not requiring intensive care. Certainly, the values may vary in other groups, but special alertness should be held in the case of this parameter. A study by Vo analysed pCO_2_ in capillary blood gas samples taken at hospital admission from 134 patients with bronchiolitis and found significantly higher mean pCO_2_ in the respiratory decompensation group (48 vs. 44 mmHg) [16]. A significant association between pCO_2_ and respiratory decompensation was shown, and an increased odds ratio was observed (OR = 1.07). We found significantly higher OR for hypercapnia in the prediction of ICU transfer, but a difference between the groups of patients needs to be addressed; while in the research by Vo over 45% of patients decompensated (and the decompensation is almost equal to ICU transfer in our study), in our group of patients the risk of ICU transfer was approximately 3% (15 out of 485 patients) [16]. The differences between the groups seem to be striking, however, the usefulness of the CBG–pCO_2_ is similar; moreover, the differences may explain different cutoff values. Furthermore, the question raised by Vo needs to be taken into consideration; a routine blood gas may not be needed in each case of bronchiolitis but may be helpful in the assessment of more severe cases, especially as a part of the more complex evaluation that includes other clinical features [16].

Our findings were in line with the study by Vo [16] also with regard to other clinical features in children with bronchiolitis. It needs to be emphasized that except for the breath rate on admission (which was higher in patients transferred to the ICU but also correlated with age and in the multiple regression model showed no relationship with the risk of an ICU transfer), there were no differences in any other clinical or laboratory baseline characteristics of children who required an intensive care unit transfer. Therefore, any parameter (pCO_2_, for example) that might be helpful in predicting the ICU transfer risk is highly needed. Furthermore, the CBG parameters do not correlate in a clinically relevant way with any of the laboratory or clinical patient’s characteristics, thus providing new information for the patient’s status assessment. An internal correlation between the CBG parameters was not strict (the highest correlation coefficient rho = −0.61 for pH–pCO_2_ correlation), suggesting that all of these parameters should be measured and interpreted independently, with no possibility of using one as a proxy for the other.

The fact that the CBG did not correlate with the length of stay may be related to many factors, including an individual response to the disease/treatment, or influence of complications (such as a bacterial suprainfection), so the CBG presented no additional value in these terms. Similarly, it did not predict antibiotic use in hospitalized children, which may be influenced by various other factors (host immunity, viral pathogenicity, suprainfections, etc.), hence, the CBG should not be considered a tool for predicting the risk or the necessity for antibiotic implementation. However, the CBG may be an auxiliary instrument in differentiating the patient’s severe status related to a primary viral infection from a severe status related to a bacterial coinfection. The first group will not potentially benefit from antibiotic treatment, and other laboratory markers might be used together with the CBG to assess the need for antibiotics.

The question of the reliability of the CBG results needs to be addressed. In the study by Yildizdas et al. (2004), a group of 116 patients aged 15 days to 160 months (mean of 56.91 months) was enrolled, and a correlation between arterial, venous, and capillary blood samples (taken without prior warming of the extremity) was assessed [7]. The study showed a strong correlation for pH, pCO_2_, BE, and HCO_3_, both for CBG–ABG and VBG–ABG and a weaker correlation for pO_2_ [7]. A coefficient of correlation (between CBG and ABG) was calculated and reached: r = 0.823 for pH, r = 0.988 for pCO_2_, r = 0.991 for BE, r = 0.991 for HCO_3_, and r = 0.674 for pO_2_. The CBG–AVG coefficients of correlation were higher compared to the VBG–ABG for all of the above parameters, except for the pH, for which it was slightly lower (r = 0.823 vs. r = 0.907). The correlation was not affected significantly even in patients with hypo/hyperthermia or capillary refill time over 3 s, only the correlation for pO_2_ disappeared in patients with hypotension [7]. Thus, the authors considered the CBG (and VBG) a useful alternative, yet, not recommending them for control of arterial pO_2_ [7]. Here, instead of analysing the pO_2_, which shows a lower reliability compared to other CBG parameters, we chose oxygen saturation to verify its correlation with pulse oximetry saturation. Nevertheless, an analysis of the pO_2_ as an independent predictor, irrespective of the CBG–ABG correlation, might add some data and verify its further usefulness. A similar compatibility was shown in a cohort of 50 children aged 14 days to 12 years [17]. The rates of agreement (with the ABG) were better for the CBG than for the VBG in the case of the pH and the pCO^2^, reaching r = 0.9024 and r = 0.9534, respectively. As in the previous study, pO_2_ showed a lower correlation (r = 0.6917) [17]. Results of seventy-five patients included in the study by Escalante-Kanashiro and Tantalean-Da-Fieno in order to establish an agreement between the CBG and the ABG showed an average correlation of r = 0.87, r = 0.86, and r = 0.65 for pH, pCO_2,_ and pO_2_, respectively [9]. The results were not affected by the confounding factors (present in 40% of children) such as hypothermia or poor perfusion, with the only exception for hypotension [9]. Correspondingly, the study by Harrison et al. (1997) conducted in 50 children over 1 month of age revealed a strong correlation between the CBG and the ABG for pH and pCO_2_ (r = 0.903, and r = 0.955, respectively), and a weaker correlation (although statistically significant) for pO_2_ (r = 0.358) [10]. It needs to be underlined that in this study capillary blood indicated lower pH (relative bias of 0.009) and higher pCO_2_ values (average bias of 0.21 kPa = 1.58 mmHg) than the arterial blood. Therefore, the risk of missing a patient in a more severe condition would be even decreased with the use of CBG, since the CBG shows acidosis and hypercapnia more frequently than the ABG. All the studies cited above were performed at paediatric intensive care units, however, in our opinion, the results may be applied to a broader group of paediatric patients, especially those hospitalized due to lower respiratory tract infections. Firstly, potential confounding factors (e.g., hypothermia and hypotension) are observed more frequently in patients who require intensive care. Secondly, patients with lower respiratory tract infections have not only been included in the ICU studies but formed a large group of cases. The cohorts of children with respiratory infections/failure were as follows: 16 patients (14% of the study group) with pneumonia and 10 with bronchiolitis (10%) in the study by Yildizdas et al. (2004) [7], 15 patients (30%) with bronchiolitis and 7 (14%) with pneumonia in the Kirubakaran study [17], 16 children (21.3%) with pneumonia in the Escalante-Kanashiro and Tantalean-Da-Fieno analysis [9], while Harrison et al. (1997) included eight patients (16%) with respiratory failure (due to an unspecified cause) [10].

The impact of the patient’s age on the CBG results seems to be negligible in the general population; a large group of 712 patients studied by Dong et al. (1985) showed that pH values are stable throughout the lifetime, while the pCO_2_ values are generally lower than in adults but comparable in infants and children aged 1 to 3 years old and then gradually start to increase to reach values of the adult population [18]. The application of the CBG may seem questionable in neonates, since studies by Courtney (1990) and Bannister (1969), performed in 77 and 13 newborns aged 1–25 days and 3–95 h of life, respectively, showed poor agreement for pCO_2_ [19,20]. A study of 50 neonates with birth asphyxia by Saili (1992) showed significant differences regarding not only pCO_2_, but also pH and pO_2_ [21]. In contrast, a study on 18 newborns (in the first 24 h of life) by MacRae and Palavradji (1966) showed clinically nonsignificant differences [22]. McLain et al. (1988) analysed 158 paired samples from a group of 41 preterm infants, including respiratory distress in 35 newborns, and concluded that pH and pCO_2_ measurements agreed on a satisfactory level [23]. The number of neonates in our series seemed to be significant (56 newborns hospitalized due to bronchiolitis), thus, we find the CBG a useful tool, at least in older newborns (here, the youngest patient was 8 days old).

Another controversy is warming the site of sampling prior to blood sampling. The results in McLain study (1988) did not depend on prior warming of the heel [23]; on the other hand, prior warming of the heel played an important role for the appropriateness of the results in the study by Gandy [8]. Nonetheless, a question of warming the site prior to sampling does not seem to play a primary role, as samples taken from warmed [24] or unwarmed finger/extremity [25,26] reflected a good correlation even in newborns. In our study, we did not perform prior warming of the site of sampling due to the difficulties in the practical verification of the degree of warming pointed out by Yildizdas et al. (2004) [7]. Moreover, a close correlation shown for a large group of patients by Yildizdas was proven in samples taken without prior warming [7].

The choice of the site for blood sampling (earlobe/fingertip/heel) may raise some doubts, but the study by Koch (1968) performed in older patients (children and adults) pointed out directly that there were no differences in the pCO_2_ agreement, regardless of the earlobe/fingertip sampling [27]. In our study, blood was taken from an unwarmed fingertip with no exceptions for newborns or preterm infants thus creating a homogeneous group.

Nevertheless, the implementation of the CBG in practical use may meet some obstacles. Although there are official local guidelines on the use of capillary blood gas sampling under some conditions (e.g., home oxygen therapy) [28,29], many physicians are hesitant to use the CBG, mainly due to the concerns on how the CBG reflects the arterial values [30,31]. On the other hand, thanks to its ease of use, and the difficulties in other monitoring methods, the CBG (capillary pCO_2_, to be exact) is being chosen as a reference method in some studies on bronchiolitis treatment, the study by Thia et al. (2008), for example, that focused on the effects of continuous positive airways pressure in bronchiolitis [32].

There are some strengths and limitations of this study. Firstly, since it was conducted in a single paediatric ward, the generalizability of our results was restricted. However, the large number of patients included in the analysis decreases the risk of biased conclusions. Secondly, due to a change of an analyser in a local laboratory the CBG parameters were calculated with two different instruments; however, no significant differences are expected and the study did not aim to compare those analysers, but focused on patients with bronchiolitis. Thirdly, due to its retrospective character, several patients (9%) did not undergo the CBG, although none of them have been transferred to the ICU. Decisions on ICU transfer were based on the patient’s status and were not influenced by the study, yet, the usefulness of a CBG-based protocol needs to be appraised in future prospective multi-centre studies. A lack of more extensive aetiology analysis is a certain limitation but we focused on the diagnosis of bronchiolitis, for which many guidelines recommend against aetiological testing.

To conclude, acidosis and hypercapnia in capillary blood gas indicate the risk of an ICU transfer, with special emphasis on pCO_2_ concentrations. Further well-controlled prospective studies on a larger scale are guaranteed and needed to support the use of CBG, and attention should also be paid to the role of repeated CBG. Given that the CBG is a much less invasive method than ABG sampling, it reflects arterial acid-base balance accurately, and presents an important value in hospitalized bronchiolitis cases, we appeal to consider the CBG as one of the options for monitoring patients with bronchiolitis.

## Figures and Tables

**Table 1:** Baseline characteristics of patients transferred and not transferred to an ICU.

	ICU-Transferred (*N* = 15)			without ICU Transfer (*N* = 470)			
	Median	LQ	UQ	Median	LQ	UQ	*p*
**age (months)**	**1.00**	**1.00**	**1.83**	**2.13**	**1.35**	**3.83**	**0.0016**
WBC (1 × 10^3^/uL)	*10.43*	*6.80*	*13.00*	*10.30*	*8.40*	*13.24*	*0.6175*
ANC (1 × 10^3^/uL)	*1.90*	*1.12*	*3.33*	*2.05*	*1.18*	*3.62*	*0.9606*
CRP (mg/L)	*1.10*	*0.52*	*2.26*	*1.00*	*0.26*	*3.57*	*0.7019*
PCT (ng/mL)	*0.12*	*0.12*	*0.13*	*0.10*	*0.07*	*0.12*	*0.1514*
**BR** (per min)	**64.50**	**62.00**	**71.00**	**60.00**	**50.00**	**64.00**	**0.0226**
HR (per min)	*146.00*	*140.00*	*162.50*	*140.00*	*132.00*	*155.00*	*0.1076*
SpO_2_ (%)	*95.00*	*92.00*	*97.00*	*96.00*	*94.00*	*97.00*	*0.4189*
pH	*7.39*	*7.37*	*7.43*	*7.41*	*7.39*	*7.43*	*0.0694*
**pCO_2_ (mmHg)**	**44.80**	**37.10**	**50.00**	**36.20**	**33.30**	**40.50**	**0.0003**
SatO_2_ (%)	*88.60*	*81.50*	*93.40*	*90.80*	*88.00*	*93.30*	*0.1798*

Median values with interquartile range, LQ—lower quartile, UQ—upper quartile; *p*-value—the U Mann–Whitney test. WBC—white blood cells, ANC—absolute neutrophil count, CRP—C-reactive protein, PCT—procalcitonin, BR—breath rate, HR—heart rate, SpO_2_—oxygen blood saturation measured by pulse oximetry. Statistically significant results are bolded, while non-significant are shown in italics.

**Table 2:** Crude and adjusted odds ratios from logistic regression models for the relationship between capillary blood gas parameters (acidosis and hypercapnia) and an ICU transfer.

	OR	95% CI		*p*	aOR	95% CI		*p*
acidosis	5.93	1.54	22.80	0.01	5.01	1.26	19.89	0.0219
hypercapnia	8.69	3.01	25.12	0.00	6.63	2.15	20.46	0.0010

OR—crude odds ratio, aOR—adjusted odds ratio, 95% CI—95% confidence interval.

**Table 3:** The matrix of correlation (Spearmann’s rank correlation coefficients rho are shown; ns—statistically non-significant).

	pH	pCO_2_	SatO_2_	Age	LOS	WBC	ANC	CRP	PCT	BR	HR	SpO_2_
pH	-	−0.61	0.36	0.15	ns	0.09	0.12	0.14	ns	ns	−0.11	0.11
pCO_2_	−0.61	-	−0.55	−0.46	ns	−0.22	−0.23	−0.11	ns	0.18	0.17	−0.24
SatO_2_	0.36	−0.55	-	0.35	ns	ns	0.14	Ns	ns	−0.15	−0.21	0.23
age	0.15	−0.46	0.35	-	ns	0.09	0.22	0.22	ns	−0.15	−0.15	ns
LOS	ns	ns	ns	ns	-	ns	ns	Ns	ns	−0.15	ns	ns
WBC	0.09	−0.22	ns	0.09	ns	-	0.68	0.34	0.17	ns	ns	0.10
ANC	0.12	−0.23	0.14	0.22	ns	0.68	-	0.56	0.33	−0.13	ns	ns
CRP	0.14	−0.11	ns	0.22	ns	0.34	0.56	-	0.36	−0.13	ns	ns
PCT	ns	ns	ns	ns	ns	0.17	0.33	0.36	-	ns	0.19	ns
BR	ns	0.18	−0.15	−0.15	−0.15	ns	−0.13	−0.13	ns	-	0.21	−0.30
HR	−0.11	0.17	−0.21	−0.15	ns	ns	ns	Ns	0.19	0.21	-	−0.18
SpO_2_	0.11	−0.24	0.23	ns	ns	0.10	ns	Ns	ns	−0.30	−0.18	-

WBC—white blood cells, ANC—absolute neutrophil count, CRP—C-reactive protein, PCT—procalcitonin, BR—breath rate, HR—heart rate, SpO_2_—oxygen blood saturation measured by pulse oximetry.

## Data Availability

Data are available on request from the authors.
